# 3-Dimensional micropillar drug screening identifies FGFR2-IIIC overexpression as a potential target in metastatic giant cell tumor

**DOI:** 10.18632/oncotarget.16883

**Published:** 2017-04-06

**Authors:** Seung Tae Kim, Jusun Kim, Sumin Shin, Sun Young Kim, Dongwoo Lee, Bosung Ku, Yong Sung Shin, Jhingook Kim, Jeeyun Lee

**Affiliations:** ^1^ Division of Hematology-Oncology, Department of Medicine, Samsung Medical Center, Sungkyunkwan University School of Medicine, Seoul, Korea; ^2^ Department of Thoracic Surgery, Samsung Medical Center, Sungkyunkwan University School of Medicine, Seoul, Korea; ^3^ Medical and Bio Device Inc. Suwon, Gyeonggi-Do, Korea; ^4^ Samsung Biomedical Research Institute, Samsung Medical Center, Sungkyunkwan University, School of Medicine, Seoul, Korea

**Keywords:** patient-derived cell (PDC), high-throughput screening (HTS), 3D culture

## Abstract

We established two patient derived tumor cells (PDCs) from right and left pulmonary metastatic lesions respectively of a patient with giant cell tumor. At that time, patient-derived tumor cells from right and left surgical specimens were collected and cultured. High-throughput screening (HTS) for 24 drugs was conducted with a micropillar/microwell chip platform using giant cell tumor PDCs. Using 6 doses per drug in 6 replicates for giant cell tumor PDCs, the dose response curves and corresponding IC50 values were calculated from the scanned images using the S+ Chip Analyzer. A sensitive response was more significantly achieved for AZD4547 (FGFR2 inhibitor) in giant cell tumor PDCs originated from the right pulmonary nodule under the micropillar/microwell chip platform using 3D culture. This sensitivity was consistent with the target expression patterns of giant cell tumor PDCs (FGFR2-IIIC mRNA expression in giant cell tumor PDCs originated from the right pulmonary nodule was increased significantly as compared to those originated from left). However, in a conventional 2D cultured MTT assay, there was no difference for IC50 values of AZD4547 between giant cell tumor PDCs originated from right and left pulmonary nodules. An HTS platform based on 3D culture on micropillar/microwell chips and PDC models could be applied as a useful preclinical tool to evaluate the intrapatient tumor/response heterogeneity. This platform based on 3D culture might reflect far better the relation between the tumor-biology and the matched targeted agent as compared to a conventional 2D cultured MTT assay.

## INTRODUCTION

Giant cell tumor of bone (GCT) is an aggressive osteolytic and potentially metastatic bone tumor, which has a predilection anatomic sites of long bones such as distal femur, proximal tibia and distal radius [1]. High recurrence rates of 18–60% following aggressive surgical resection have been reported for GCT, which rarely undergoes malignant transformation [2–5]. However, distant metastasis of GCTs of the bone is extremely rare. Lung metastasis of a benign GCT of the bone was first reported in 1926. One of the most common organs for distant metastasis from GCT is lung, ranging from 1 to 9% in incidence [6–9].

Fibroblast growth factor receptor (FGFR) is a transmembrane tyrosine kinase receptor that mediates angiogenesis, as well as proliferation and survival of cancer cells [10, 11]. Dysregulation of the FGFR gene expression, including gene amplification and mutation, is known to be associated with cancer progression [10, 12]. The role of fibroblast growth factor (FGF) receptor 2 IIIC (FGFR2 IIIC) has been underscored in GCT, especially in osteogenic differentiation of GCT cells [13]. In GCT, FGF-2 stimulates FGFR2 expression, resulting in increased alkaline phosphatase (ALP) and osteopontin expression. Inhibition of FGFR2 through siRNA decreased the expression of osteopontin in GCT stromal cells resulting in FGF2 ligand induced downstream signaling pathway, and possibly cell growth inhibition [13].

We previously demonstrated that patient derived tumor cells (PDCs) are feasible preclinical model which closely resemble the patient tumor genome and clinical response [14]. The micropillar/microwell chip platform has been developed to facilitate miniaturized 3D cell cultures and perform high throughput, biochemical and cell-based assays [15]. Owing to the importance of microenvironment, especially in GCTs where both stromal and tumor cells are important components of the tumor, we utilized 3-D HTS to screen for potentially efficacious anti-cancer drug in metastatic GCT PDC model.

## RESULTS

### 3D cell cultured HTS platform using PDC models derived from a patient with metastatic giant cell tumor

**Figure 1:**
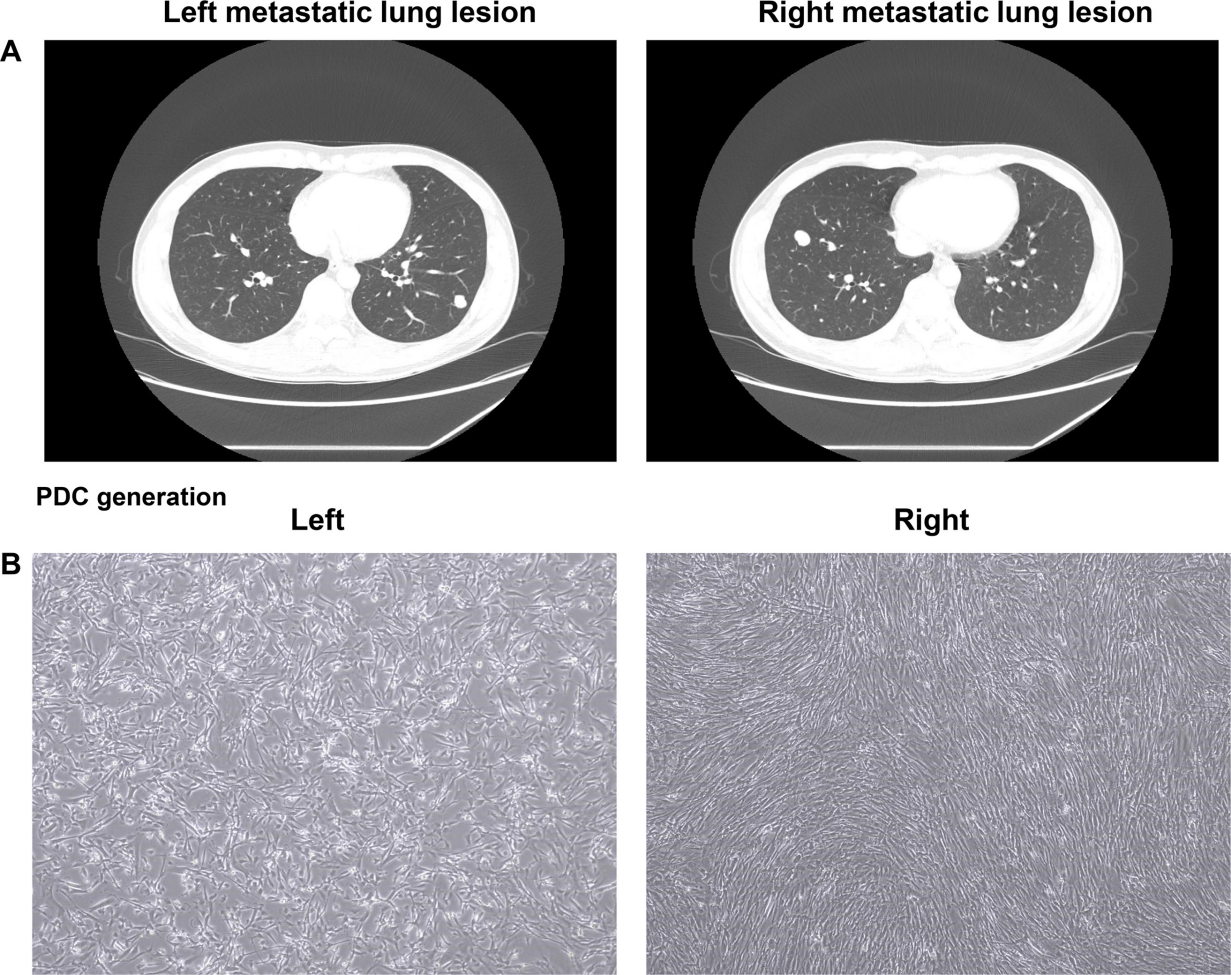
CT scan of the chest in giant cell tumor patient with left and right metastatic lung lesions (**A**). PDCs originated from left and right metastatic lung lesions, respectively (**B**).

**Figure 2:**
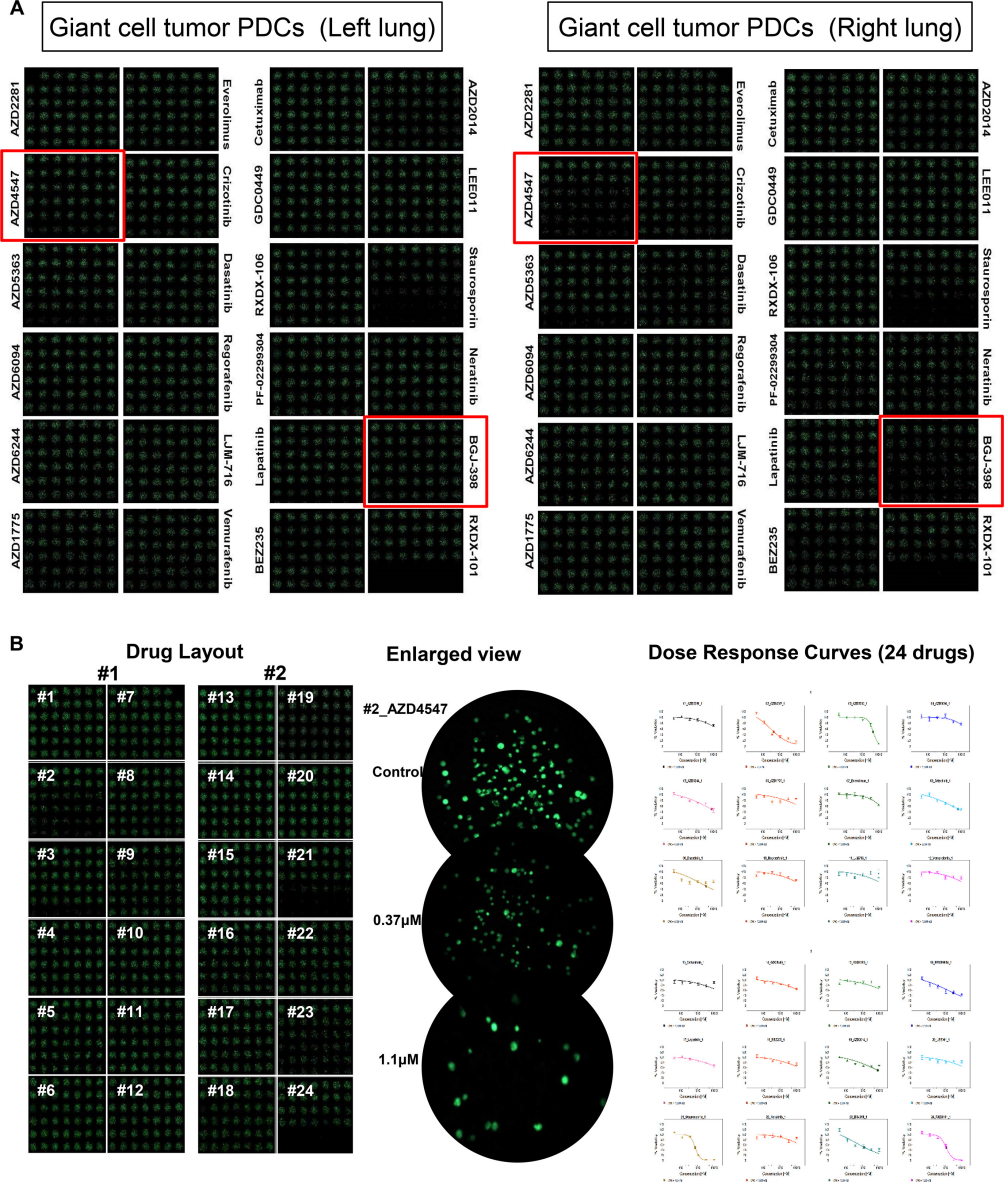

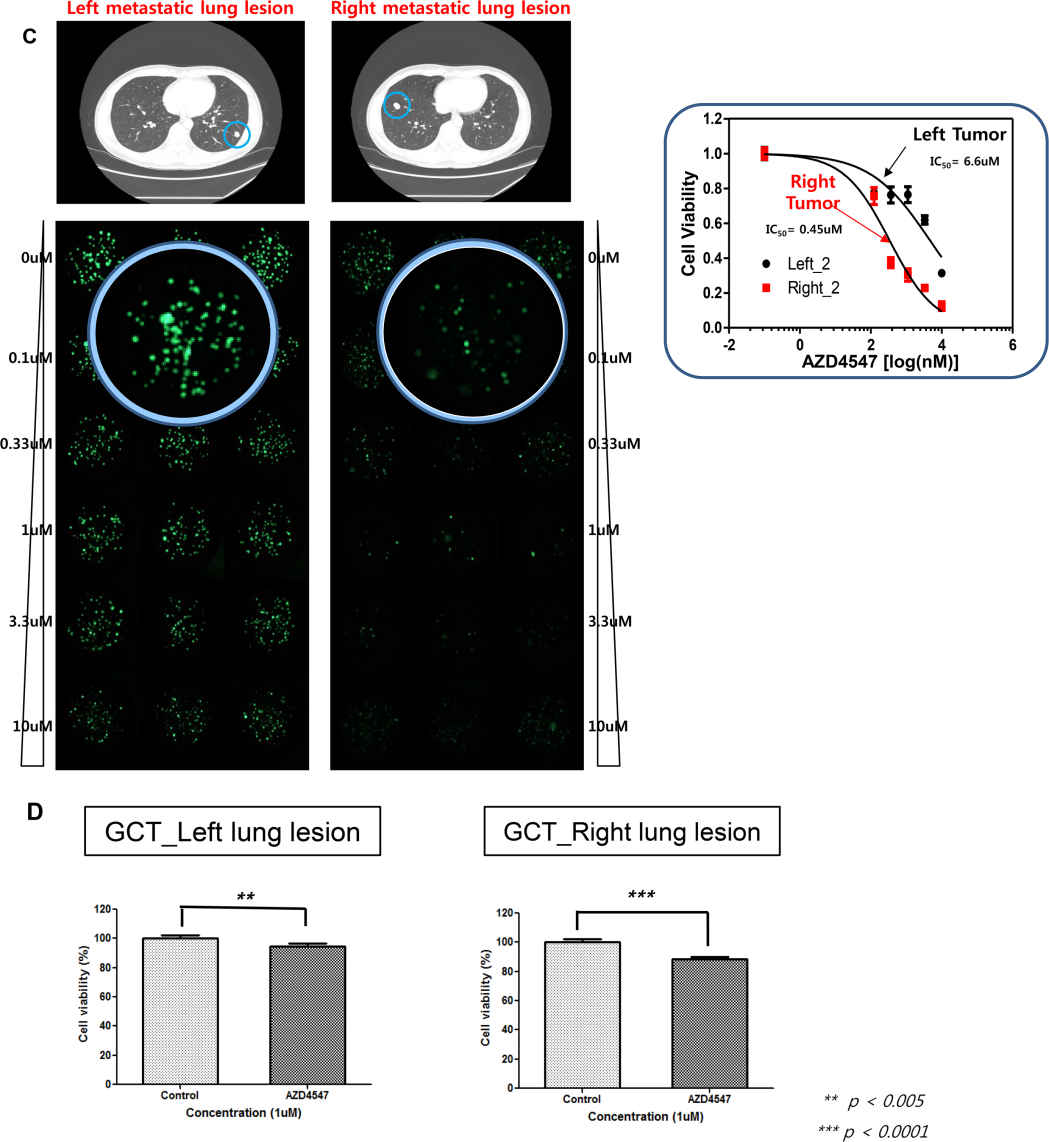
3D cell cultured HTS platform using PDCs derived from left and right metastatic lung lesions, respectively (**A**). A sensitive response by corresponding IC50 values of AZD4547 in giant cell tumor PDCs originated from the right pulmonary nodule under the micropillar/microwell chip platform using 3D culture as compared to left (**B** and **C**). The cell proliferation assay in conventional monolayer culture using MTT assay (**D**).

A 30-year-old man with a recurrent giant cell tumor received sacrectomy, coccygectomy and pelvic mass removal followed by postoperative radiotherapy 4 years ago. During regular follow-up, a chest computed tomography (CT) scan revealed two right and left pulmonary nodules (Figure 1A). The patient underwent metastasectomy for the right and left pulmonary nodules. At that time, patient-derived tumor cells from right and left surgical specimens were collected and cultured (Figure 1B). Cells were grown in 3D culture condition with alginate to be screened using micropillar/microwell chip system. 24 drugs (AZD2281, AZD4547, AZD5363, AZD6094, AZD6244, AZD1775, everolimus, crizotinib, dasatinib, regorafenib, LJM-716, vemurafenib, cetuximab, GDC0449, RXDX-106, PF-02299304, lapatinib, BEZ235, AZD2014, LEE011, staurosporin, neratinib, BGJ-398 were tested (Figure 2A). Based on the image analysis of the 3D cell culture HTS platform using PDCs, among 24 drugs tested AZD4547 and BGJ-398 demonstrated more significant growth inhibition of PDCs from the right pulmonary metastatic lesion as compared to left. Using 6 doses per drug in 6 replicates for right and left giant cell tumor PDCs, the dose response curves and corresponding IC50 values were calculated from the scanned images using the S+ Chip Analyzer. A sensitive response by corresponding IC50 values was also more significantly achieved for AZD4547 (FGFR2 inhibitor) and BGJ-398 (pan-FGFR inhibitor) in giant cell tumor PDCs originated from the right pulmonary nodule under the micropillar/microwell chip platform using 3D culture as compared to left (Figure 2B and 2C). The cell proliferation assay was also confirmed in monolayer culture using MTT assay (Figure 2D).

### Differential expression of FGFR2 IIIc between right and lung metastatic nodules

**Figure 3:**
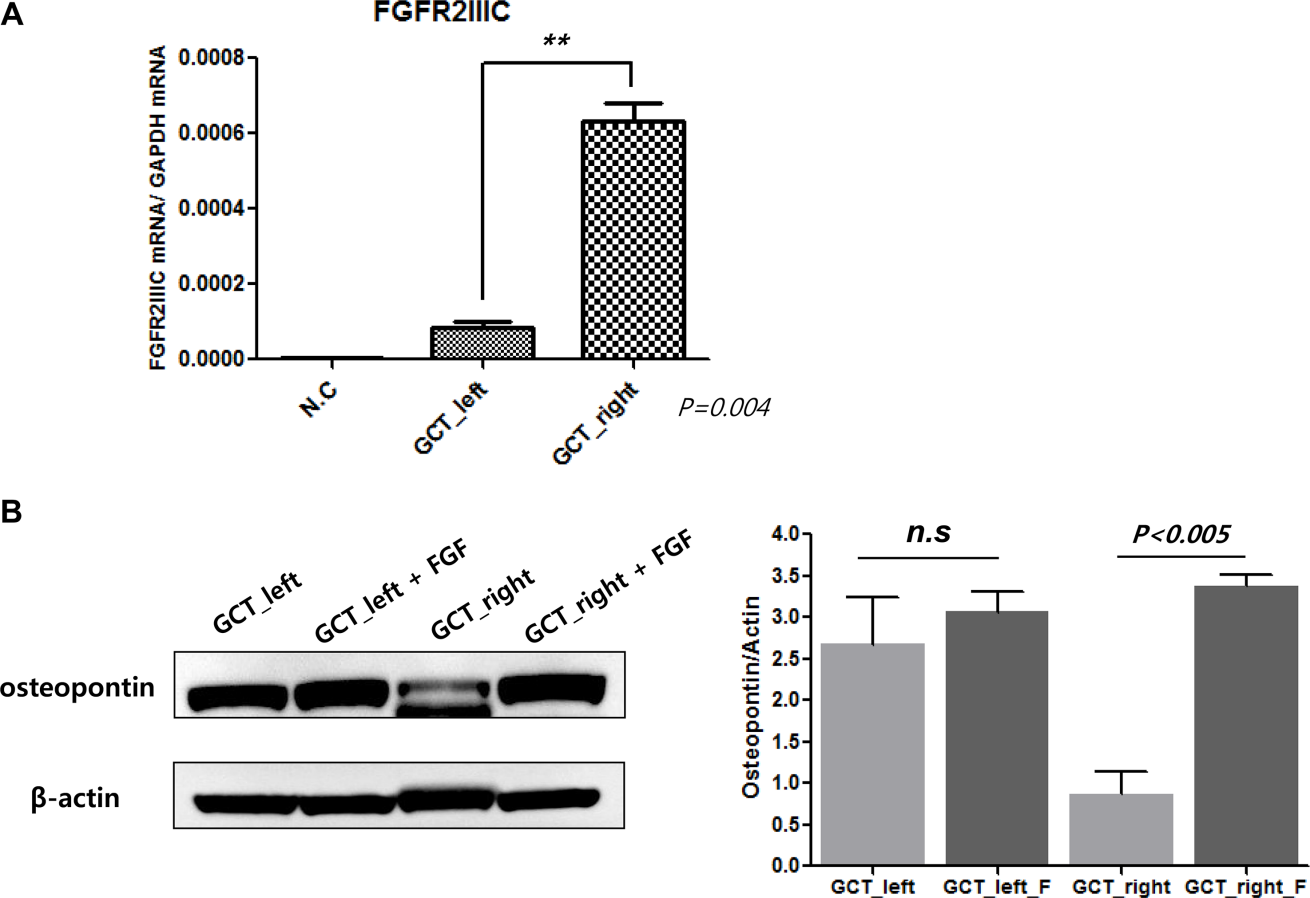
mRNA expression of FGFR2 IIIC in PDCs from left and right metastatic lung lesions, respectively (**A**). Baseline osteopontin level and changes of osteopontin by fibroblast growth factor (FGF) in PDCs from left and right metastatic lung lesions, respectively (**B**).

In order to investigate the response heterogeneity to AZD4547 (FGFR2 inhibitor) and BGJ-398 (pan-FGFR inhibitor) in the HTS with a micropillar/microwell chip platform using 3D culture between giant cell tumor PDCs originated from right and left, we measured relative mRNA expression of FGFR2 IIIC, as target of FGFR inhibitor, via qRT-PCR. As shown in Figure 3A, FGFR2IIIC expression was significantly increased in PDCs originated from right pulmonary nodule as compared to PDCs originated from left. Moreover, we evaluated the difference of osteogenic differentiation between giant cell tumor PDCs originated from right and left. Osteopontin (an osteoblastic marker) protein level was increased in giant cell tumor PDCs originated from left [13]. However, fibroblast growth factor (FGF) treatment promoted the osteopontin protein level in giant cell tumor PDCs established from right which was sensitive to FGFR inhibitor but not in PDCs generated from left nodule (Figure 3B).

## DISCUSSION

In this study, we have demonstrated the effectiveness of a new experimental preclinical platform for high-throughput screening (HTS) of multiple drugs combining patient-derived tumor cell (PDC) models and a micropillar/microwell chip assay based on 3D cultures to evaluate intrapatient tumor /response heterogeneity. HTS for 24 drugs was conducted with a micropillar/microwell chip platform using giant cell tumor PDCs originated from right and left metastatic nodules, respectively. Using 6 doses per drug in 6 replicates for right and left giant cell tumor PDCs, the dose response curves and corresponding IC50 values for 24 drugs were calculated from the scanned images using the S+ Chip Analyzer. A sensitive response was more significantly achieved for AZD4547 (FGFR2 inhibitor) in giant cell tumor PDCs originated from the right pulmonary nodule under the micropillar/microwell chip platform using 3D culture. This sensitivity was consistent with the target expression patterns of giant cell tumor PDCs (FGFR2IIIC mRNA expression in giant cell tumor PDCs originated from the right pulmonary nodule was increased significantly as compared to those originated from left.). Our analysis suggests that PDCs are useful preclinical models and HTS-platform with 3D cultured cells on the micropillar/microwell chip using PDCs might be applied to evaluate intrapatient tumor /response heterogeneity.

We compared the outcomes between the HTS-platform with 3D cultured cells on the micropillar/microwell chip and a conventional MTT assay with 2D cultured cells. For PDC models derived from right and left metastatic nodules of a patient with giant cell tumor, the IC50 values of AZD4547 were evaluated using these two methods. The IC50 values of drugs tested against 3D cultured PDCs on the chip could divide two PDC models (right vs. left) according to molecular heterogeneity. However, a conventional MTT assay with 2D cultured cells didn't draw the response heterogeneity to drug for two PDC models (right vs. left) according to molecular heterogeneity. This discrepancy may be caused by the difference of reflections for tumor heterogeneity, microenvironments, and phamacogenomic information between 2D- and 3D-cultured cells. The difference in IC50 values between 3D and 2D cell culture systems has already been reported and is considered a distinctive characteristic of cells cultured in 3D systems [16, 17]. Culturing human primary cells from patients under conditions more similar to those *in vivo*, such as 3D microenvironments, can potentially provide valuable insight into *in vivo* drug efficacy as the 3D cell cultures can maintain specific biomedical and morphological features that resemble those of the corresponding tumor. Conventional HTS has been based on two-dimensional (2D) cell monolayer cultures [18]. The formation of tumor-like 3D structures is strongly inhibited in 2D monolayer cultures by the strong affinity of cells for most artificial surfaces and the restriction to a 2D space. Thus, the results of HTS based on 3D cell cultures might reflect far better the relation between the tumor-biology and the matched targeted agent as compared to a conventional 2D cultured MTT assay.

In addition to interpatient response heterogeneity, understanding intrapatient response heterogeneity is also essential for designing effective therapeutic strategies in the era of precision medicine [19]. The functional impact of intrapatient heterogeneity on cancer remains to be fully understood. Intrapatient heterogeneity influences the dynamic tumor landscape and plays a key role in shaping response to specific therapies [20, 21]. The difference in sensitivity to specific drugs between PDCs originating from right and left metastatic lesions may be caused by intertumor heterogeneity, i.e., the presence of different genetic alterations in different metastatic tumors from a single patient (Figure 3). With the advent of therapies targeting specific oncogenes, it is possible to use mutation detection strategies aimed at these oncogenes to assess tumor specimens for intertumor heterogeneity. Such heterogeneity is potentially important because it has been shown to affect responses to molecularly targeted treatments in cancers. Although we clearly recognize the significance of intertumor heterogeneity for realizing precision medicine, it is not easy to overcome it. It is impossible to perform molecular profiling of multiple metastatic lesions from the same patient to evaluate the presence of intertumoral heterogeneity because of the high cost. Thus, a HTS platform based on 3D culture of PDCs on micropillar/microwell chips might be a useful approach for evaluating the presence of intertumoral heterogeneity. To the best of our knowledge, this is the first study to evaluate the intrapatient tumor/response heterogeneity through HTS on the 3D cell culture micropillar/microwell chip platform using PDCs.

Giant cell tumor is an aggressive bone tumor consisting of multinucleated osteoclast-like giants cells and proliferating osteoblast-like stromal cells. Previous study using giant cell stromal cells from patient specimens demonstrated that FGFR2 signaling plays an essential role in bone development and promotes differentiation of immature osteoblastic cells [13]. They specifically showed that FGFR2-IIIC overexpression in GCT stromal cells was correlated with activation of FGF signaling pathway leading to osteoblastic differentiation. As shown in Figure 3, only right lung nodule had FGFR2 IIIC overexpression whereas FGFR2 IIIC was not overexpressed in left lung nodule. Osteopontin, a marker for osteoblastic differentiation, was increased by FGF ligand only in right lung. Hence, the sensitivity to FGFR2 inhibitor may be related to baseline FGFR2 IIIC expression level and possibly related to baseline osteoblastic differentiation level in each nodule which is aligned with previous report [13].

In conclusion, An HTS platform based on 3D culture on micropillar/microwell chips and PDC models could be applied as a useful preclinical tool to evaluate the intrapatient tumor/response heterogeneity. This platform based on 3D culture might reflect far better the relation between the tumor-biology and the matched targeted agent as compared to a conventional 2D cultured MTT assay.

## MATERIALS AND METHODS

### Cell lines and patient-derived cell (PDC) culture

With informed consent form, giant cell tumor samples were obtained from right and left pulmonary metastatic lesions of a single patient. Collected tissue was minced and dissociated by enzymatic methods. Right and left giant cell tumor patient-derived cells (PDCs) were cultured. The cells were grown in RPMI 1640 supplemented with 10% fetal bovine serum (FBS; Gibco BRL, Paisley, UK) and 1% antibiotic-anti-mycotic solution (Gibco BRL). The medium was changed every 3 days, and cells were maintained at 37°C in a humidified 5% CO_2_ incubator. PDCs were passaged using TrypLE Express (Gibco BRL) to detach cells when they reached 80–90% confluence.

### DNA/RNA extractions

Cultured cells (passage 1 to 2) were harvested with TrypLE Express. Genomic DNA was isolated using a QIAamp DNA Mini Kit (Qiagen, GmBH, Hilden, Germany) and total RNA was isolated with an RNeasy Mini Kit (Qiagen) according to the manufacturer's instructions. The concentrations of genomic DNA and RNA were measured using a NanoDrop ND-100 (Nano Drop Technologies, Wilmington, DE, USA). Genomic DNA and RNA were stored at −80°C.

### High-throughput screening (HTS) of PDCs originated from right and left metastatic pulmonary nodules in the same patient on a micropillar/microwell chip platform

Sixteen cultured two giant cell tumor PDCs (Right vs. Left) were seeded into 3D culture media consisting of DMEM F/12 containing 1% antibiotic-antimycotic solution, 10 mM HEPES, B27, N2, Glutamax (Gibco BRL), 10 nM human gastrin I, 1 mM N-acetyl-L-cysteine (Sigma Aldrich), 10 μg/ml insulin, 20 ng/ml bFGF, and 50 ng/ml EGF (PeproTech, Rocky Hill, NJ, USA). After 3–5 days, the tissues were dissociated into single cells using accutase (Gibco BRL) and prepared for loading on the micropillar chip. The chip layout is designed for screening 12 compounds in a single micropillar/microwell chip, as previously described [15]. In the micropillar chip, approximately 100 cells are immobilized with 0.5% alginate (Sigma Aldrich) in each micropillar. In this study, we tested 24 compounds (AZD2281, AZD4547, AZD5363, AZD6094, AZD6244, AZD1775, everolimus, crizotinib, dasatinib, regorafenib, LJM-716, vemurafenib, cetuximab, GDC0449, RXDX-106, PF-02299304, lapatinib, BEZ235, AZD2014, LEE011, staurosporin, neratinib, BGJ-398) with 6 replicates per sample in two giant cell tumor PDCs (Right vs. Left), respectively. A 40 nl droplet of cell/alginate mixture and 950 nl droplet of media or compounds were dispensed with a S+ microarrayer (S+ Chip Scanner, Samsung Electro-Mechanics Co., Ltd, South Korea). After incubation for 1 day, the micropillar chip containing cells was transferred to a new microwell chip filled with various test compounds and the combined chips were incubated for 7 days to test compound efficacy as described previously [15].

### Quantitative real-time reverse transcription polymerase chain reaction

Total RNA was isolated from cells using the Qiagen RNeasy Mini Kit protocol (Qiagen, Valencia, CA, USA) according to the manufactures’ recommendations. Total RNA from each sample was reverse transcribed with the SuperScript^®^III First-Strand Synthesis System using Oligo(Td) 20 primer (Invitrogen Corp, Carlsbad, California, USA). All qPCR reactions were performed with a 7500 Fast Real-Time PCR System (Applied Biosystems, Foster City, CA), using FGFR2IIIC primer Forward: 5′CGCTGGGGAATATACGTCGTGCT-3′ and FGFR2IIIC primer reverse: 5′-CTGGACTCAGCCG AAACTGT-3′. The reaction produced a 297 bp PCR product. Glyceraldehyde-3-phosphate-dehydrogenase (GAPDH) was used as the housekeeping gene for relative quantification. All genes were run in triplicate to allow for the assessment of technical variability.

### Cell treatment and viability assay

After pathologic confirmation, cells were seeded at a density of 1–2 × 10^6^ cells/10-mm dish or 5,000 cells/well in 96-well plates for immunoblot analysis and cell proliferation assays and treated for 3–5 days with various doses of drugs, as indicated. Inhibition of cell proliferation was determined using Cell Titer Glo (Promega, Madison, WI, USA) according to the manufacturer's protocol.

### Immunoblot analysis

Total proteins from GC cell lines and PDCs were isolated using RIPA buffer (Sigma-Aldrich, St. Louis, MO, USA) containing a protease inhibitor cocktail (Roche, Mannheim, Germany) and phosphatase inhibitor cocktail (Roche), and protein concentrations were determined using a Quick Start Bradford Protein Assay (Bio-Rad, Hercules, CA, USA). Aliquots containing 30 μg of protein were subjected to 10% SDS-polyacrylamide gel electrophoresis and electrotransferred to nitocellulose membranes. The membranes were blocked with 5% nonfat dry milk in Tris-buffered saline containing 0.1% v/v Tween 20, and probed overnight at 4°C with specific antibodies. Horseradish peroxidase-conjugated anti-rabbit or mouse IgG (Vector, Burlingame, CA, USA) was used as a secondary antibody, and signals were detected by chemiluminescence using ECL Western Blotting Substrate (Thermo Scientific, Rockford, IL, USA) and visualized using LAS-4000 (Fujifilm, Tokyo, Japan). We evaluated the expression of specific molecular markers such as FGFR2, osteopontin corresponding to two PDCs (Right vs. Left) used in the micropillar/microwell chip platform.

## References

[1] Turcotte RE, Ferrone M, Isler MH, Wong C (2009). Outcomes in patients with popliteal sarcomas. Can J Surg.

[2] Ghert M, Alsaleh K, Farrokhyar F, Colterjohn N (2007). Outcomes of an anatomically based approach to metastatic disease of the acetabulum. Clin Orthop Relat Res.

[3] Goldring SR, Schiller AL, Mankin HJ, Dayer JM, Krane SM (1986). Characterization of cells from human giant cell tumors of bone. Clin Orthop Relat Res.

[4] Katz E, Nyska M, Okon E, Zajicek G, Robin G (1987). Growth rate analysis of lung metastases from histologically benign giant cell tumor of bone. Cancer.

[5] McDonald DJ, Sim FH, McLeod RA, Dahlin DC (1986). Giant-cell tumor of bone. J Bone Joint Surg Am.

[6] Viswanathan S, Jambhekar NA (2010). Metastatic giant cell tumor of bone: are there associated factors and best treatment modalities?. Clin Orthop Relat Res.

[7] Kay RM, Eckardt JJ, Seeger LL, Mirra JM, Hak DJ (1994). Pulmonary metastasis of benign giant cell tumor of bone. Six histologically confirmed cases, including one of spontaneous regression. Clin Orthop Relat Res.

[8] Osaka S, Toriyama M, Taira K, Sano S, Saotome K (1997). Analysis of giant cell tumor of bone with pulmonary metastases. Clin Orthop Relat Res.

[9] Tubbs WS, Brown LR, Beabout JW, Rock MG, Unni KK (1992). Benign giant-cell tumor of bone with pulmonary metastases: clinical findings and radiologic appearance of metastases in 13 cases. AJR Am J Roentgenol.

[10] Turner N, Grose R (2010). Fibroblast growth factor signalling: from development to cancer. Nat Rev Cancer.

[11] Brooks AN, Kilgour E, Smith PD (2012). Molecular pathways: fibroblast growth factor signaling: a new therapeutic opportunity in cancer. Clin Cancer Res.

[12] Jang JH, Shin KH, Park JG (2001). Mutations in fibroblast growth factor receptor 2 and fibroblast growth factor receptor 3 genes associated with human gastric and colorectal cancers. Cancer Res.

[13] Singh S, Singh M, Mak IW, Turcotte R, Ghert M (2012). Investigation of FGFR2-IIIC signaling via FGF-2 ligand for advancing GCT stromal cell differentiation. PLoS One.

[14] Lee JY, Kim SY, Park C, Kim NK, Jang J, Park K, Yi JH, Hong M, Ahn T, Rath O, Schueler J, Kim ST, Do IG (2015). Patient-derived cell models as preclinical tools for genome-directed targeted therapy. Oncotarget.

[15] Lee DW, Choi YS, Seo YJ, Lee MY, Jeon SY, Ku B, Kim S, Yi SH, Nam DH (2014). High-throughput screening (HTS) of anticancer drug efficacy on a micropillar/microwell chip platform. Anal Chem.

[16] Hidalgo M, Amant F, Biankin AV, Budinska E, Byrne AT, Caldas C, Clarke RB, de Jong S, Jonkers J, Maelandsmo GM, Roman-Roman S, Seoane J, Trusolino L (2014). Patient-derived xenograft models: an emerging platform for translational cancer research. Cancer Discov.

[17] Wong HL, Wang MX, Cheung PT, Yao KM, Chan BP (2007). A 3D collagen microsphere culture system for GDNF-secreting HEK293 cells with enhanced protein productivity. Biomaterials.

[18] Shoemaker RH (2006). The NCI60 human tumour cell line anticancer drug screen. Nat Rev Cancer.

[19] Seoane J, De Mattos-Arruda L (2014). The challenge of intratumour heterogeneity in precision medicine. J Intern Med.

[20] Fridman WH, Pages F, Sautes-Fridman C, Galon J (2012). The immune contexture in human tumours: impact on clinical outcome. Nat Rev Cancer.

[21] Junttila MR, de Sauvage FJ (2013). Influence of tumour micro-environment heterogeneity on therapeutic response. Nature.

